# Molecular characterization of mutations associated with resistance to second-line tuberculosis drug among multidrug-resistant tuberculosis patients from high prevalence tuberculosis city in Morocco

**DOI:** 10.1186/s12879-018-3009-9

**Published:** 2018-02-27

**Authors:** Amal Oudghiri, Hind Karimi, Fouad Chetioui, Fathiah Zakham, Jamal Eddine Bourkadi, My Driss Elmessaoudi, Amin Laglaoui, Imane Chaoui, Mohammed El Mzibri

**Affiliations:** 1 Unité de Biologie et Recherches Médicales, Centre National de l’Energie, des Sciences et Techniques Nucléaires, BP 1382 RP, 10001 Rabat, Morocco; 2 Equipe de Recherche en Biotechnologies et Génie des Biomolécules, Faculté des Sciences et Techniques de Tanger, Ancienne Route de l’Aéroport, Km 10, Ziaten, BP 416, Tanger, Morocco; 3 Laboratoire de la Tuberculose, Institut Pasteur du Maroc, Casablanca, 1 Place Louis Pasteur, Boulevard Abdelmoumen, 20250 Casablanca, Morocco; 4 Service de Pneumo-Phtisiologie, Hôpital Moulay Youssef, CHU Rabat, Avenue Sidi Mohamed Ben Abdallah, Al Akkari, Rabat, Morocco

**Keywords:** Tuberculosis, Multi-drug resistance, Extensively drug resistance, Drug susceptibility testing, Genetic mutations, Morocco

## Abstract

**Background:**

The emergence of extensively drug-resistant tuberculosis (XDR-TB) has raised public health concern for global TB control. Although multi drug-resistant tuberculosis (MDR- TB) prevalence and associated genetic mutations in Morocco are well documented, scarce information on XDR TB is available. Hence, the evaluation of pre-XDR and XDR prevalence, as well as the mutation status of *gyrA*, *gyrB*, *rrs*, *tlyA* genes and *eis* promoter region, associated with resistance to second line drugs, is of great value for better management of M/XDR TB in Morocco.

**Objectives:**

To evaluate pre-XDR and XDR prevalence, as well as the mutation status of *gyrA*, *gyrB*, *rrs*, *tlyA* genes and *eis* promoter region, associated with resistance to second line drug resistance, in 703 clinical isolates from TB patients recruited in Casablanca, and to assess the usefulness of molecular tools in clinical laboratories for better management of M/XDR TB in Morocco.

**Methods:**

Drug susceptibility testing (DST) was performed by the proportional method for first line drugs, and then the selected MDR isolates were tested for second line drugs (Ofloxacin, Kanamycin, Amikacin and Capreomycin). Along with DST, all samples were subjected to *rpoB*, *katG* and *p-inhA* mutation analysis by PCR and DNA sequencing. MDR isolates as well as 30 pan-susceptible strains were subjected to PCR and DNA sequencing of *gyrA*, *gyrB*, *rrs*, *tlyA* genes and eis promoter, associated with resistance to fluoroquinolones and injectable drugs.

**Results:**

Among the 703 analysed strains, 12.8% were MDR; Ser531Leu and Ser315Thr being the most common recorded mutations within *rpoB* and *katG* genes associated with RIF and INH resistance respectively. Drug susceptibility testing for second line drugs showed that among the 90 MDR strains, 22.2% (20/90) were resistant to OFX, 2.22% (2/90) to KAN, 3.33% (3/90) to AMK and 1.11% (1/90) to CAP. Genotypic analysis revealed that 19 MDR strains harbored mutations in the *gyrA* gene; the most recorded mutation being Asp91Ala accounting for 47.6% (10/21), and 2 isolates harbored mutations in the promoter region of *eis* gene. No mutation was found in *gyrB*, *rrs* and *tlyA* genes. Moreover, none of the pan-susceptible isolates displayed mutations in targeted genes.

**Conclusion:**

Most of mutations associated with SLD resistance occurred in *gyrA* gene (codons 90-94) and *eis* promoter region. These findings highlight the impact of mutations in *gyrA* on the development of fluroquinolones resistance and provide the first estimates of the proportion of pre-XDR-TB among MDR-TB cases in Morocco.

## Background

Worldwide, tuberculosis (TB) remains one of the most infectious diseases and leading cause of high mortality and morbidity [1]. The situation is even made worse by the emergence of drug-resistant strains, especially multidrug resistant strains (MDR) defined as resistant to at least isoniazid (INH) and rifampin (RIF), which poses a real threat to the success of TB control programs worldwide [2]. During the last decade, a bleaker picture emerged with the discovery of the extreme form of drug resistance. Extensively drug-resistant (XDR) TB, defined as MDR with resistance to fluoroquinolones (FQs) and at least one of the three injectable second- line drugs (amikacin (AMK), kanamycin (KAN), and capreomycin (CAP)), has emerged and represents a great health problem for national TB programs around the world. Recent data showed that at least one case of XDR TB was reported by 117 countries by the end of 2015 [3]**.**

Yet, when considering the burden of MDR and XDR in a given population, a significant but often overlooked category is “pre-XDR”, described as MDR-TB isolates with additional resistance to either a FQ or one of the three second-line injectable drugs, but not both [4].

In Morocco, the global incidence of tuberculosis is very high, with nearly 107 new cases per 100,000 inhabitants yearly, representing about 28,000 new cases per year. Global surveys revealed that the rate of MDR strains is 1% and 8.7% among new cases and re-treated patients respectively [3].

In Morocco, as it’s the case in almost developing countries, diagnosis of TB is mainly based on direct smear examination that lacks sensitivity, and bacterial culture on Lowenstein–Jensen (L/J) medium which is time consuming, and drug susceptibility testing (DST), which has long turnaround times, is done only on request and is limited to first line drugs.

Since the discovery of the association between mutations affecting the function and/or expression of chromosome-encoded targets and resistance to anti-tuberculosis drugs, molecular based methods have emerged as alternative tools for rapid, sensitive, faster and accurate diagnosis of TB and evaluation of the resistance status of the bacteria. Resistance to RIF and INH is well documented. The latter is mainly due to point mutations in *rpoB* gene for RIF and in *katG* and *ahpC* genes and the promoter region of *inhA* gene for INH. The main mechanism of resistance to FQs is point mutations in the *gyrA* and *gyrB* genes, encoding the two subunits of the DNA gyrase. Most mutations conferring resistance to FQs occur in a short segment termed the Quinolone Resistance Determining Region (QRDR) in the *gyrA* gene and less frequently in *gyrB*. In the *gyrA* gene, mutations are mostly found in codons 90 and 94, and rarely in codons 88 and 91, whereas for *gyrB* the codons affected are mainly 472 and 510 [5].

Point mutations in the *rrs* gene, encoding the 16S rRNA subunit, are associated with resistance to injectable drugs: CAP, AMK and KAN, and are located especially between nucleotides 1400 and 1500 [6–10]. Of particular interest, 3 SNPs are mainly found in the *rrs* gene at positions 1401, 1402 and 1484, and displaying different resistance patterns [11]. Additionally, *tlyA* gene, encoding the 2′-O-methyltransferase that modifies nucleotide C1409 in helix 44 of 16S rRNA and nucleotide C1920 in helix 69 of 23S rRNA is also associated with resistance to CAP [12].

Resistance to AMK could also be a result of mutations in the promoter region of *eis* gene, known to enhance the intracellular survival of a related bacterium [13].

FQs and/or injectable aminoglycosides are often used in the treatment of bacterial infection other than TB. Therefore, exposure to these drugs may contribute to evolution of resistance of TB patients to these agents [14].

In Morocco, like other developing countries, and due to constraint in resources and high TB burden, resistance to FQs and injectable drugs is not tested routinely. Moreover, scarce informations are available with regard to the presence of XDR strains in Morocco [3] and mutations associated with resistance to FQs and injectable drugs. Therefore, we have planned to evaluate pre-XDR and XDR prevalence, as well as the mutation status of *gyrA*, *gyrB*, *rrs*, *tlyA* genes and *eis* promoter region, associated with resistance to second line drugs (SLDs), in 703 clinical isolates from TB patients recruited in the highest TB incidence region in Morocco. The aim of this study was to assess the usefulness of these molecular tools in clinical laboratories for better management of M/XDR TB in Morocco.

## Methods

### Clinical isolates

This prospective study was conducted between 2010 and 2012. Clinical samples were collected in Casablanca, a city with the highest TB incidence in Morocco (139.8 per 100.000 population annually), including almost one-fifth of the total cases of TB recorded in the country according to National Anti-tuberculosis Program, Department of Health, Rabat, Morocco [15] and harboring the highest number of drug-resistant TB namely MDR-TB cases [3]. Moreover, the Reference TB Laboratory in Pasteur Institute in Casablanca receives mainly patients with failure treatment and relapses, and therefore is the appropriate Center to have enough MDR cases for SLDs resistance testing.

A total of 703 clinical isolates were collected from TB confirmed pulmonary patients and sent to Mycobacterial Laboratory at Pasteur Institute in Casablanca for direct examination according to Ziehl-Neelsen method and bacterial culture on L/J medium.

For each recruited patient, a questionnaire including, personal information, socio-demographic characteristics and previous treatment history, was completed.

The study protocol was approved by the Ethical Committee for Pasteur Institute of Casablanca, and written informed consent was obtained from each study subject.

### Drug susceptibility testing

Cultures obtained on L/J medium were collected and then tested for DST to first line drugs: RIF and INH; and second line drugs: Ofloxacin (OFX), AMK, KAN and CAP. DST was performed using the proportional method with L/J medium [16]. The critical drug concentrations were 0.2 μg/ml for INH, 40 μg/ml for RIF, 2 μg/ml for OFX, 40 μg/ml for KAN, AMK and CAP each. The critical proportion of resistant bacillus necessary to define a resistant strain is 1% for all tested drugs [17].

### *Mycobacterium tuberculosis* DNA isolation

Scraped bacterial colonies were recovered in 400 μl of distilled water and boiled at 100 °C for 10 min to inactivate bacteria and release the mycobacterial DNA [18, 19]. Recovered DNA was immediately used for PCR amplification or stored at − 20 °C until use.

### PCR amplification

Amplification of drug-resistant genes; *rpoB*, *katG*, *inhA*, *gyrA*, *gyrB*, *rrs*, *eis* and *tlyA*; was carried out by PCR using specific primers reported in Table 1.

**Table 1:** Primer sequences and positions used to amplify the relevant genes

ATB drug	Gene	Primer	Sequence	Tm	Length
RIF	*rpoB*	TR 8	5’-TGCACGTCGCGGACCTCCA-3’	58 °C	157 bp
		TR 9	5’-TCGCCGCGATCAAGGAGT-3’		
INH	*katG*	RTB 59	5’-TGGCCGCGGCGGTCGACATT-3’	62 °C	419 bp
		RTB 36	5’-GGTCAGTGGCCAGCATCGTC-3’		
	*inhA*	inhA P5	5’-CGCAGCCAGGGCCTCGCTG-3’	60 °C	246 bp
		inhA P3	5’-CTCCGGTAACCAGGACTGA-3’		
FQs	*gyrA*	gyrA F	5’-TGACATCGAGCAGGAGATGC-3’	59 °C	320 bp
		gyrA R	5’-GGGCTTCGGTGTACCTCATC-3’		
	*gyrB*	gyrB F	5’-GTGGAAATATGTTGGCCGTC-3’	58 °C	413 bp
		gyrB R	5’-GTCGTTGTGAACAACGCTGTG-3’		
Aminoglycosides /cyclic peptides	*rrs*	rrs F	5’-GTAATCGCAGATCAGCAACG-3’	58 °C	216 bp
		rrs R	5’-GTGATCCAGCCGCACCTT-3’		
	*tlyA*	tlyA F	5’-GTCTCTGGCCGAACTCGAAG-3’	52 °C	1000 bp
		tlyA R	5’-ATTGTCGCCCAATACTTTTTCTAC-3’		
	*eis*	eis F	5′-AAA TTC GTC GCT GAT TCT CG-3’	56 °C	387 bp
		eis R	5’-CGC GAC GAA ACT GAG ACC-3’		

*ATB* Anti-tuberculosis, *F* forward, *R* reverse, *bp* base pair

Amplification reactions were performed in a total volume of 25 μl. The amplification mixture contained 0.5 mM of each primer, 2.5 mM of each dNTP (dATP, dCTP, dGTP and dTTP), 25 mM MgCl_2_ and 1 unit of Hotstar Taq DNA polymerase (Invitrogen) and 2 μl of crude DNA sample in 1× Taq polymerase buffer. For each reaction, a negative control in which DNA template was omitted from the amplification mixture, and a positive control containing DNA from H37Rv strain, were included.

For all genes, PCR amplification was started by a denaturation at 94 °C for 7 min. Then, forty cycles of PCR were performed with denaturation at 94 °C for 1 min, primer annealing for 1 min at the corresponding Tm and primer extension for 1 min at 72 °C. At the end of the last cycle, the mixture was incubated at 72 °C for 7 min. Amplicons were visualized after electrophoretic fractionation in 1 to 2% agarose gel in 0.5 X TBE buffer and staining with ethidium bromide.

### Sequencing reaction

Amplified fragments were firstly purified using the illustra ExoProStar1-Step (GE Healthcare Life Sciences). Direct sequencing of amplicons was performed using Big Dye Terminator kit (version 3.1) (Applied Biosystem, Foster City, CA, USA) that includes dideoxynucleotides labelled with four fluorochromes of different colours. For each PCR product, both strands were sequenced, in independent reactions, using the mentioned above primers. The resulting chromatograms were manually edited to ensure sequence accuracy and added to the alignment component of MEGA 5 software.

## Results

A total of 703 TB patients were recruited from Pasteur Institute in Casablanca. According to demographic data, the mean age of patients was 33.38 (SD: 9.7) with extreme ages of 3 and 62, 80% of TB patients were in the age group of 16-45 years old. The sex ratio was 3.2.

Patients were clinically categorized according to WHO guidelines [20]; 32.4% (228/703) of TB patients were new cases, 12.4% (87/703) failed treatment, 25.2% (177/703) were relapsed and 4.7% (33/703) of patients were under treatment after loss to follow patients. The information regarding 25.3% (178/703) of patients was not available.

DST was performed for RIF and INH as first line drugs. Of the 703 strains, 68.6% (482/703) were RIF and/or INH resistant strains. Indeed, 28.2% (198/703) of strains were RIF mono-resistant, 27.6% (194/703) were INH mono-resistant and 12.8% (90/703) were MDR strains.

The 90 phenotypic MDR strains were selected for further analyses, including genotypic resistance and second line drug susceptibility testing. Genotyping resistance results of the 90 MDR strains are reported in Table 2 and revealed that 84 strains were genotypic MDR strains harboring mutations in *rpoB* and, *katG* gene or *inhA* gene promoter; Ser531Leu and Ser315Thr being the most common recorded mutations within *rpoB* and *katG* genes respectively.

**Table 2:** Frequency of mutations identified by sequencing in the *rpoB*, *katG* genes, and *inhA* gene promoter of MTB isolates

Gene	Position	Type of Mutation	Amino acid change	Number of isolates	Total
*rpoB*	513	CAA → CCA	Glu / Pro	1	84
	513	CAA → CTA	Glu/ Leu	1	
	516	GAC → GTC	Asp / Val	3	
	516	GAC → TAC	Asp / Tyr	2	
	516	GAC → CAC	Asp / His	3	
	526	CAC → CGC	His / Arg	3	
	526	CAC → AGC	His / Ser	4	
	531	TCG → TGG	Ser / Trp	5	
	531	TCG → TTG	Ser / Leu	62	
*katG*	315	AGC → ACC	Ser / Thr	81	
*inhA promoter*	-15	C → T		3	

DST for SLDs showed that among the 90 MDR strains, 22.2% (20/90) were resistant to OFX, 2.22% (2/90) to KAN, 3.33% (3/90) to AMK and 1.11% (1/90) to CAP.

Genotypic analysis of *gyrA*, *gyrB*, *rrs*, *tlyA* and *eis* was performed on all the 90 MDR strains, as well as 30 pan-susceptible MTB isolates, results are reported in Tables 3 and 4. Overall, 19 MDR strains harbored mutations in the *gyrA* gene and 2 strains in the promoter region of *eis* gene (Table 3). No mutation was found in *gyrB*, *rrs* and *tlyA* genes.

**Table 3:** Frequency of mutations associated with SLDs resistance in MDR MTB isolates

ATB drug	Gene	Position	Substitution	Amino acid change	Number of isolates	Sub-total	Total
FQs	*gyrA*	Codon 90	GCG → GTG	Ala / Val	2	19	21
			GCG → ACG	Ala / Thr	2		
		Codon 91	TCG → CCG	Ser / Pro	3		
		Codon 94	GAC → GCC	Asp / Ala	10		
			GAC → AAC	Asp / Asn	1		
			GAC → CAC	Asp / His	1		
	*gyrB*	–	–	–	0	0	
Aminoglycosides /cyclic peptides	*rrs*	–	–	–	0	0	
	*tlyA*	–	–	–	0		
	*eis*	Position 12	C → T	–	1	2	
		Position 14	C → T	–	1

**Table 4:** Frequency of mutations associated with polymorphism in MDR TB isolates

ATB drug	Gene	Position	Substitution	Amino acid change	Number of isolates
FQs	*gyrA*	Codon 95	AGC → ACC	Ser / Thr	20
	*gyrB*		–	–	–
Aminoglycosides /cyclic peptides	*rrs*		–	–	–
	*tlyA*	Position 33	A → G	–	90
	*eis*		–	–	0

Of the 20 phenotypic FQs (OFX) resistant strains, 19 were identified by DNA sequencing to display various mutations in the QRDR region of *gyrA* gene. Among the 70 susceptible isolates to OFX, 69 were found to have no mutation in the *gyrA* and *gyrB* QRDR and were considered to be wild type and one strain harbored a mutation at position 90 (Ala90Val) in *gyrA* gene.

Our results showed that the most recorded mutation in the QRDR region of *gyrA* gene is the substitution of GAC > GCC at codon 94 (Asp91Ala) accounting for 47.6% (10/21). Other point mutations were found: TCG > CCG (Ser91Pro) in 3 cases (14.3%), GCG > GTG (Ala90Val) and GCG > ACG (Ala90Thr) in 2 strains each (9.52%), and GAC > AAC (Asp94Asn) and GAC > CAC (Asp94His) in 1 strain each (4.8%). Interestingly, no strain harbored more than one amino acid change.

Among the 6 strains phenotypically resistant to Aminoglycosides/cyclic peptides (AMK, *n* = 3; KAN, *n* = 2; and CAP, *n* = 1), only 2 exhibited mutations in the promoter region of *eis* gene, these 2 strains are phenotypically resistant to AMK, 1 strain harbored mutation at position 12 (C > T) and another at position 14 (C > T). No mutation was found in *rrs* and *tlyA* genes. None of the pan-susceptible isolates displayed mutations in targeted genes.

Of particular interest, all genotypic pre-XDR strains, harboring mutation in *gyrA* or *eis* promoter genes, belong to the 84 genotypic MDR strains.

Other mutations considered as genetic polymorphisms and known to be not associated with drug resistance were also reported, including AGC > ACC at the codon 95 (Ser95Thr) of *gyrA* gene occurred in 20 MDR cases and A > G at position 33 of *tlyA* gene and did not confer any amino acid change. The A33G was found in all MDR isolates as well as in the 30 pan-susceptible strains.

## Discussion

It is widely accepted that the knowledge of the *Mycobacterium tuberculosis* (MTB) resistance spectrum is important for the effective treatment of MDR- and XDR-TB. In Morocco, many studies have already investigated first line drug resistance prevalence and associated genetic mutations [21–24]. However, to our best knowledge, no study has explored the pre-XDR and XDR TB status in Morocco. Therefore, we have planned through this study to investigate the prevalence of second line drug resistance and to identify the main resistance-related mutations in the MTB strains from TB patients residing in hot spot area of TB.

Among the 703 recruited strains, 90 cases were MDR (12.8%), which is in agreement with previously reported data in Morocco [3, 25]. DNA genotyping showed that mutations in RRDR region of *rpoB* gene and, in *katG* gene and *inhA* promoter region, are the main SNPs conferring resistance to RIF and INH, respectively, confirming previously reported data [21–23] and reaffirming the usefulness of *rpoB*, *katG* and *inhA* promoter mutations as predictive markers for MDR TB. Moreover, molecular analysis could be limited to rpoB516, rpoB526, rpoB531, katG315 and inhA− 15 mutations, due to their high sensitivity and spectificity, to detect MDR strains.

Many studies have reported that in vitro testing is particularly cumbersome and difficult to interpret for second-line drugs, and therefore a growing interest in rapid molecular detection methods for resistance to these drugs has been well documented [11]. In this study, DST was performed only for OFX, KAN, AMK and CAP, the results showed that among the 90 MDR strains, 26 (28.9%) were resistant to one SLD, and no XDR strain was found. Molecular analysis showed that 19 strains have point mutations in *gyrA* gene and only 2 strains in the promoter region of *eis* gene.

FQs are the mainstay of treatment for patients with MDR and XDR TB since their inclusion in therapeutic regimens improves treatment outcome [26]. However, resistance to FQs increases the risk of failure and relapse, and hinders the success of national TB programs.

In this study, 22 strains were resistant to OFX, the only FQ tested in our TB laboratory, giving a rise to high frequency of pre-XDR strains. Molecular analysis showed that 19 strains harbored mutation in the QRDR region of *gyrA* gene and no mutation in *gyrB* gene was observed. Overall, 6 point mutations were observed and the most common one was the D94A reported in 10 cases. Previous data reported worldwide, showed that the codon 94 is the most common mutated one, but the main mutation reported was D94G [27]. D94A is associated with low level of FQ resistance in contrast to D94G which is thought to provide the greatest advantage for the cell with regard to increased resistance and the minimum loss of fitness [28]. The polymorphism S95 T in *gyrA* gene was reported in previous studies as a marker of the evolutionary history of the organism and does not correlate with drug resistance [29, 30]. In this study, S95 T was reported in 22.22% of MDR isolates, suggesting that the corresponding strains belong to Principal Genetic Group 1 or 2 of the M*.* tuberculosis complex.

Two FQs resistant isolates had no mutation neither in the QRDR of either *gyrA* nor in *gyrB* genes. Therefore, it is relevant to screen for mutations outside the QRDR region of *gyrA* and in other genes, such as mfpA (Rv3361c), or the active efflux pump RV2686c-Rv2687c-Rv2688c operon, associated with FQs resistance [31–33].

One strain that harbored mutation in the QRDR of *gyrA* gene was phenotypically sensitive to OFX. This discordance could be due to DST error, possibly resulting from undetected hetero-resistance.

Of particular interest, molecular approach, using DNA sequencing, have high sensitivity to detect FQ resistance (90%). Our results are in agreement with previously reported results in Germany (90.6%) [34], in France (87.5%) [11], in India (81%) and in South Africa (79%) [35]. However, slightly lower prevalence was reported in China (74.5%) [31] and in Vietnam (75.6%) [36]. Other countries exhibit low prevalence like Philippines (32%) and Moldova (55%) [37].

The QRDR region of *gyrA* is well defined and the majority of mutations found in this region confer resistance to FQs, albeit not at the same level [5]. In contrast, mutations in *gyrB* gene are associated with low level of FQ resistance and occur less frequently than in *gyrA* gene, even though it’s widely recommended to consider the *gyrB* gene mutation status when screening for XDR MTB strains [5, 27, 37, 38]. However, in Morocco, and due to limited resources and high drug resistance TB, sequencing of the *gyrB* gene might not be a great concern for determination of FQ resistance.

Worldwide, the high prevalence of pre-XDR TB as well as the occurrence of FQ resistance within pan-susceptible isolates might imply the inappropriate use of these drugs [39–41]. Indeed, FQs antibiotics (Ofloxacin, ciprofloxacin, and levofloxacin) are among the most potent SLDs used for treatment of MDR-TB. However, FQ resistant *M. tuberculosis* strains results from injudicious use of this class of drugs either in the management of MDR-TB or by excess use of these drugs in the treatment of respiratory tract infections as well as other bacterial infections, increasing the burden of selective pressure and compromising their efficacy in the treatment of TB [42, 43]**.**

For injectable drugs, DST was performed for AMK, KAN and CAP, and clearly showed that resistance to these drugs is rare, and only few strains exhibited a resistant status. In the present study, genetic mutations, associated with injectable drugs resistance, were investigated in all 90 MDR MTB strains, despite their susceptible/resistance profiles to SLDs, and in the 30 pan-susceptible strains. SNPs associated resistance were not detected neither in *rrs* nor in *tlyA* genes.

It is widely accepted that mutations within *tlyA* gene, resulting in CAP resistance, are rare and represent less than 3% of phenotypically resistant strains [12]. However, when reported, they were not found in any CAP susceptible strains, making them potentially highly specific markers of CAP resistance [44]. Of particular interest, investigation of the *tlyA* gene SNP revealed an A33G substitution, without any amino acid changes (Leu33Leu). This polymorphism was recently reported by Sowajassatakul et al. [45] and was found in all MDR MTB strains as well as pan-susceptible strains, suggesting a clonal selection of MDR TB in Morocoo and could be used for phylogenetic studies of MDR TB strains.

Mutations in the *rrs* gene, encoding the 16S rRNA bacterial subunit, cause high-level resistance to KAN and cross-resistance to AMK and sometimes CAP [33], and the more prevalent mutation (A1401G), has been reported as a surrogate marker for high-level resistance to KAN and AMK [46–48]. The absence of mutations in the *rrs* gene among our MDR strains could be probably due to limited number of resistant isolates to injectable drugs.

Mutations within *eis* promoter region were reported to be largely associated with KAN resistance [6, 13]. In the present study, the *eis* promoter SNP C-12 T was found in 1/2 (50%) of AMK resistant strains and SNP C-14 T occurred in 1/3 (33.33%) of KAN resistant strains, and none of the KAN and AMK sensitive strains harbored any mutation in the *eis* promoter region. These SNPs are very common and were considered as non-specific markers of KAN and/ou AMK resistance, as they are reported in both resistant and sensitive strains.

Available data related to mutations associated with resistance to SLD confirm that a single mutation, or even a set of mutations in a single gene, does not adequately predict phenotypic resistance to AMK, KAN and CAP. It is likely that a combination of different gene mutations for each injectable drug will be needed to best predict phenotypic resistance [9]. Based on these results, special attention is given to high rate of FQs resistance within MDR-TB and the possible reduced efficacy of drug combinations including FQs in treating MDR-TB. Unlikely, DST for SLDs is still out of reach for many low income countries that are placing MDR-TB patients on empirical treatment with a FQ without knowing the susceptible/resistance status. Available molecular tests mainly genotype MTBDR*sl* would add a great value for detection of SLDs resistance particularly FQs [11].

MDR-TB and XDR-TB are indicators of TB control failures; they emerge due to several reasons: (1) insufficient drug regiments for TB cases; (2) no adherence to an appropriate regimen; (3) poor quality of drugs; and (4) transmission of MDR-TB and XDR-TB in the community. The lack of data on XDR-TB trends in Morocco is mainly due to the lack of routine DST for second line anti-TB drugs.

There were no XDR strains in this collection. To the best of our knowledge, XDR MTB strains are rare in Morocco [3], but the high proportion of pre XDR-MTB strains is of concern. In this field, the identification of pre-XDR TB will be of a great interest for clinicians to monitor closely TB patients and forestall the progression to XDR TB which is more difficult to treat and is often associated with poor treatment success and has the potential to severely cripple global control efforts for TB.

The present study is very informative and give for the first time data on genetic mutations associated with resistance to second line drugs in Morocco. However, the main limitation was the small number of resistant strains to injectable drugs which may not reflect the real burden of drug resistance, especially XDR, in Morocco.

## Conclusion

Most of mutations associated with SLD resistance occurred in *gyrA* gene (codons 90-94) and *eis* promoter region. Other genes namely *gyrB*, *rrs* and *tlyA* did not harbor any mutations associated with resistance to SLDs. These findings highlight the impact of mutations in *gyrA* on the development of FQ resistance and provide the first estimates of the proportion of pre-XDR-TB among MDR-TB cases in Morocco. Therefore, MDR TB strains must be systematically screened for *gyrA* mutations, for rapid detection of second-line TB drug resistance which is of a great interest for appropriate treatment regimens and for limiting spread of drug resistant TB.

## Abbreviations

AMK: Amikacin
CAP: Capreomycin
DR-TB: Drug resistant tuberculosis
DST: Drug susceptibility testing
FLDs: First line drugs
FQ: Fluoroquinolones
HIV: Human immunodeficiency virus
INH: Isoniazid
KAN: kanamycin
L/J: Lowenstein Jensen
MDR: Multidrug resistant
MTB: *Mycobacterium tuberculosis*
OFX: Ofloxacin
PCR: Polymerase chain reaction
Pre-XDR: Pre-extensively drug resistant
QRDR: Quinolone resistance determining region
RIF: Rifampin
SLDs: Second line drugs
SNP: Single nucleotide polymorphism
TB: Tuberculosis
TBE: Tris-Borate- EDTA
WHO: World Health Organization
XDR: Extensively drug resistant
